# Twenty-six years of involvement with cystic echinococcosis: a case report

**DOI:** 10.1186/s13256-021-02810-9

**Published:** 2021-05-12

**Authors:** Hosein Safari, Somayeh Mirzavand, Abdollah Rafiei, Molouk Beiromvand

**Affiliations:** 1 Department of Neurosurgery, Golestan Hospital–Ahvaz Jundishapur University of Medical Sciences, Ahvaz, Khuzestan Iran; 2 Department of Parasitology, School of Medicine, Ahvaz Jundishapur University of Medical Sciences, P.O. Box 61357–15794, Ahvaz, Khuzestan Iran; 3 Infectious and Tropical Diseases Research Center, Health Research Institute, Ahvaz Jundishapur University of Medical Sciences, Ahvaz, Khuzestan Iran

**Keywords:** *Echinococcus granulosus*, Spinal, Cystic echinococcosis

## Abstract

**Introduction:**

Spinal hydatidosis, a zoonotic disease caused by infection with* Echinococcus* spp. larvae, is rare, but its treatment remains a significant medical challenge. Approximately 70% of patients with spinal hydatidosis have lesions in their liver, 0–15% have lung involvement, and only 0.5–2% have bone involvement.

**Case presentation:**

Here we report a 38-year-old Iranian man with spinal hydatidosis, who had a history of eight times surgery in over of 26 years due to hydatid cyst in the liver, lungs, and chest wall. At the most recent admission to hospital he presented with chest pain, paraplegia, and urinary incontinence. Magnetic resonance imaging revealed thoracic spinal hydatid disease. He underwent surgery, and the hydatid cysts were completely removed. Lower extremity forces recovered dramatically and completely within 4 weeks.

**Conclusion:**

Spinal hydatidosis is a rare disease, but it is associated with a high degree of morbidity, mortality, and poor prognosis. Because of the infiltrative nature of hydatid disease, surgery alone is rarely curative. The current case study demonstrates the importance of a suitable surgical approach, adequate intraoperative prophylaxis to prevent cyst rupture, and prolonged complete paraplegia.

## Introduction

Human cystic echinococcosis (CE), caused by the larval form of *Echinococcus granulosus sensu stricto* is a parasitic zoonotic disease which mainly occurs in pastoral areas worldwide [1–3]. Lesions are localized to the liver in approximately 70% of patients with CE and to the lungs in approximately 20%; other cases show the involvement of other organs [1]. Bone involvement is seen in 0.5−2% of hydatid cases, with most of these cases involving hydatid cysts in the spine.

## Case presentation

A 38-year-old Iranian man, residing in a rural area, was admitted to our surgery unit with a history of back pain, chest pain, paraplegia and urinary incontinence within the last 45 days. The patient had a history of hydatid cyst(s) in the liver, lungs, and chest wall. The first surgery occurred 26 years previously when he was 12 years old and underwent thoracotomy for two hydatid cysts in the left lung. Subsequently, in April 1999, the patient was diagnosed with a hydatid cyst in his left lung and underwent surgery again. Nine years later, after confirmation of a hydatid cyst in his left lung, he underwent thoracotomy. In May 2011, computerized tomography (CT) revealed multiple cysts located behind the left lung and the fourth rib (R4), leading to the diagnosis of paraspinal hydatidosis and a second thoracotomy. In May 2014, his imaging results demonstrated the presence of four hydatid cysts in the left hemithorax. Total cystectomy was performed for one cyst under the latissimus dorsi, and two cysts behind the third rib (R3) and one cyst behind his left clavicle were drained. In the same year, one hydatid cyst was detected in his liver, and hepatic resection was performed. In March 2018, imaging results demonstrated the presence of multiple cystic lesions under R3, R3 and the fifth rib (R5). Thoracotomy was once again performed, and the cystic lesions and the necks of R3, R4, and R5 were removed. After surgery, albendazole therapy (400 mg/kg) was initiated and continued.

**Fig. 1:**
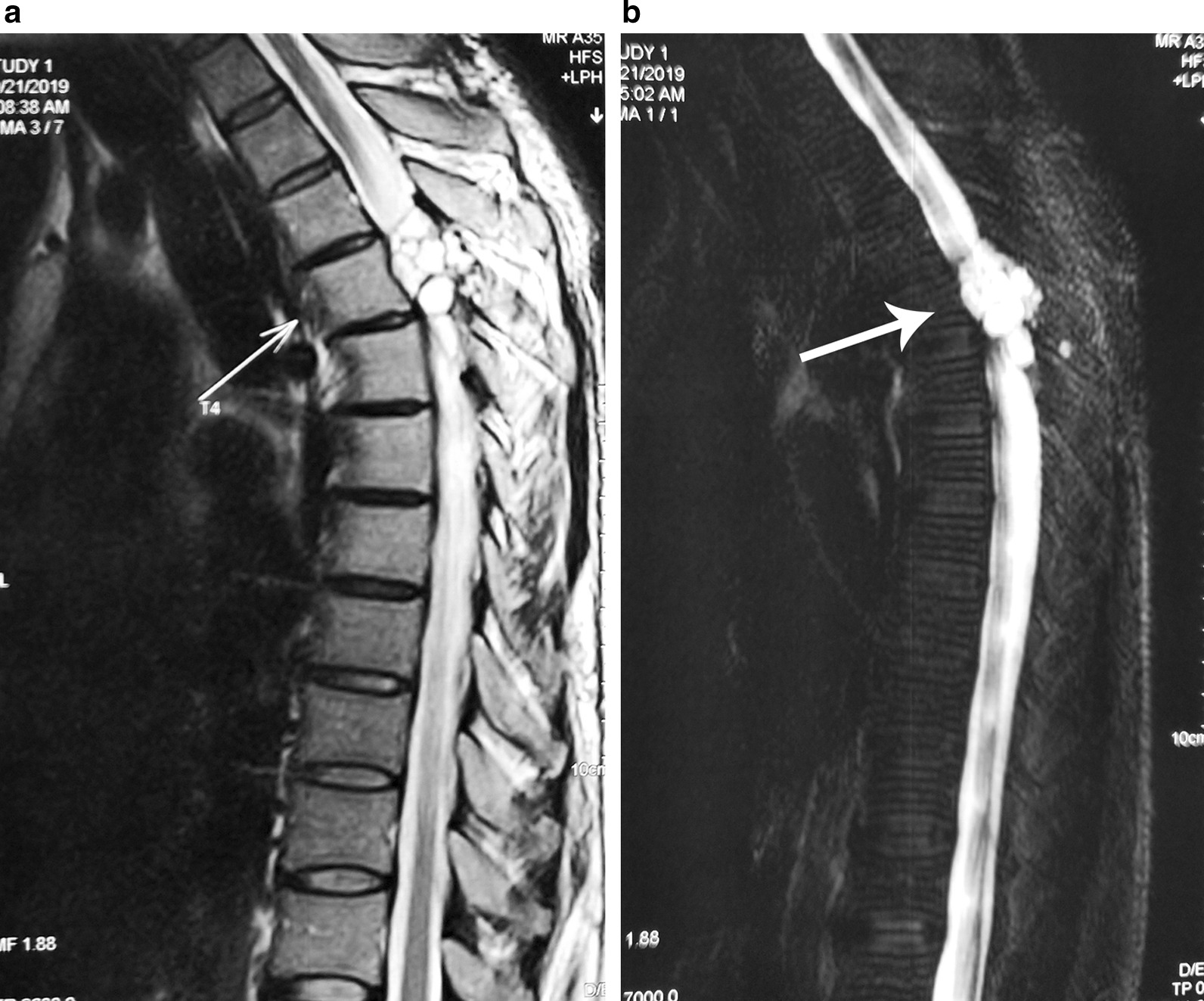
**a** Sagittal magnetic resonance imaging image at the level of the second thoracic vertebra of the patient, showing a lesion consisting of multiple cysts (white arrow) in the thoracic spinal cord. **b** Two-dimensional myelogram image showing a multiple cystic lesion (white arrow)

**Fig. 2:**
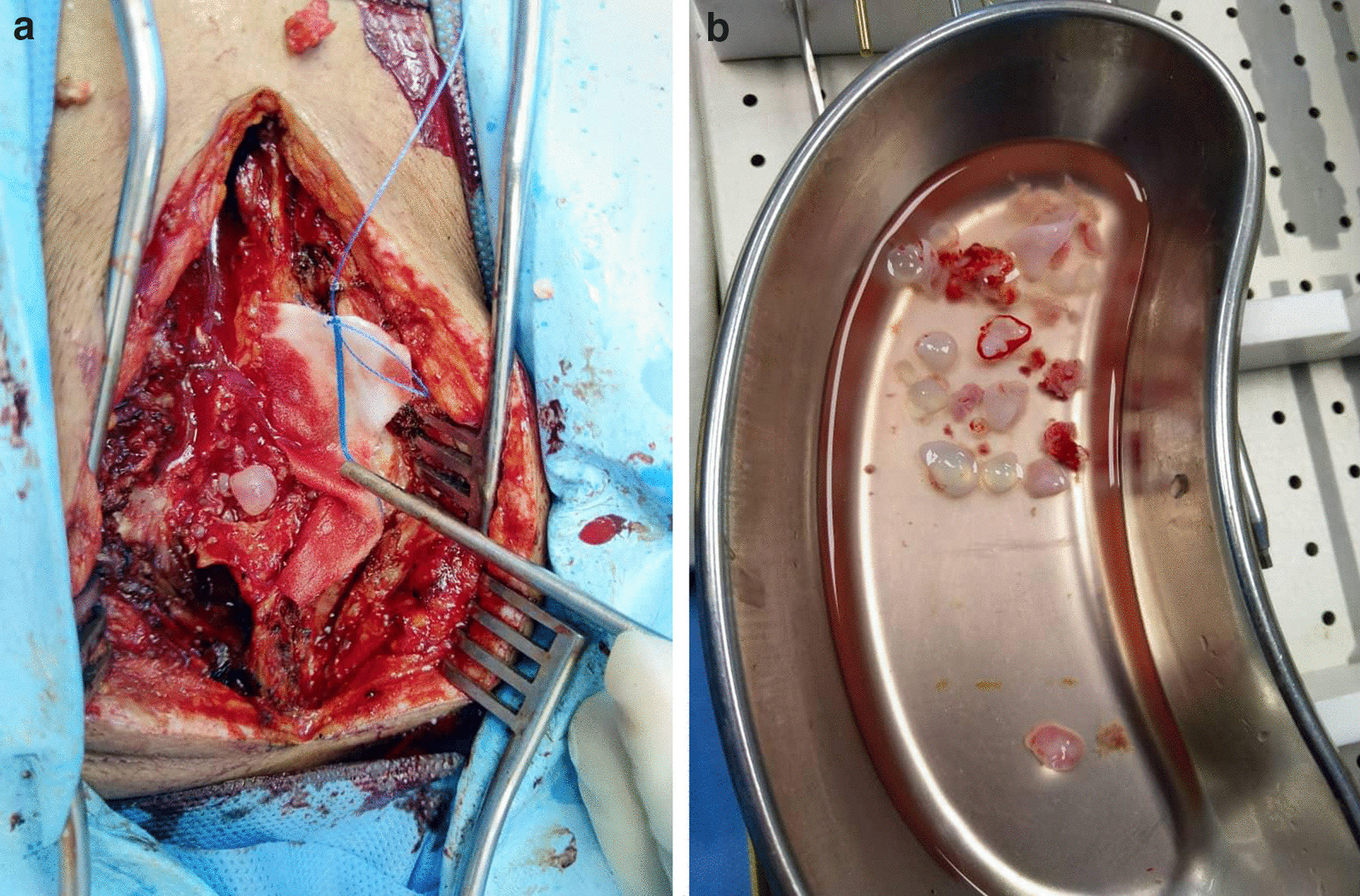
**a** Intraoperative photos of the patient. **b** Multiple grape-like daughter cysts, which were completely removed by surgical resection with the costotransverse approach

In December 2019 the patient was referred to our surgery unit with progressive weakness. Spinal magnetic resonance imaging (MRI) showed multiple spinal epidural cystic lesions at the level of the third to fourth thoracic vertebrae (T3–T4) (Figs. 1, 2) and that the pedicles on both sides of T4, some parts of the lamina, and the vertebral body were destroyed. The patient underwent surgical resection with the costotransverse approach, and multiple epidural cystic lesions at the T3–T4 level were completely removed. Multiple extradural cystic lesions were carefully excised to avoid intraoperative rupture of the cysts. Intraoperatively, irrigation with hypertonic saline (as scolicidal agents) and cotton pads soaked with hypertonic saline were used. Since the T3 and T4 pedicles had been destroyed, posterior fusion with pedicular screw was performed. The diagnosis of hydatid cyst was confirmed by pathological examination, following which treatment with 400 mg/kg albendazole was started, with the recommendation that the treatment continue for 6 months. Within 2 weeks after surgery, his lower extremity forces dramatically returned and he was full force after 4 weeks.

## Discussion and conclusions

Spinal hydatidosis is a rare form of hydatidosis and affects fewer than 1% of all patients with hydatidosis. Approximately one half of all patients with hydatidosis of bone have spinal hydatidosis; the other 50% have hydatidosis in the thoracic region. Previous studies have demonstrated that approximately 85% and 25–77% of patients suffered from back pain and paraplegia, respectively [4]. In our case, the lesion was extradural and located in the thoracic region (T3–T4), with paraspinal extension, and the patient had a history of back pain, chest pain, urinary incontinence, and paraplegia.

Surgical treatment with removal of the whole cyst(s) is the gold standard treatment in spinal hydatidosis [5]. Several factors, such as location of the cyst, familiarity with the surgical approach, and surgeon preference, are involved in the choice of surgical procedures [6]. Moreover, choosing the suitable surgical technique depends on an accurate diagnosis of the hydatid cysts in order to prevent intraoperative cyst spillage. Surgery in thoracic cases is mainly posterior [4]. During the surgery, most surgeons use scolicidal agents, such as hypertonic saline, 0.5% silver nitrate, chlorhexidine, and/or 80% ethanol to prevent rupture of hydatid cysts. Among these, 3% hypertonic saline is the most frequently used scolicidal agent [4].

Primary hydatid cysts usually contain daughter cysts, and the rupture of these daughter cysts can lead to secondary cysts [7]. The risk of release of the daughter cysts increases with bone involvement. Although it was not easy to distinguish primary or secondary cysts in the present case, the patient’s history suggests the possibility of secondary cysts. Despite a history of 1-month complete paraplegia, a dramatic neurologic recovery was observed, and thepatient fully recovered within 4 weeks. This finding suggests the slow growth nature of the lesion.

Because of the invasive nature of hydatid disease, surgery alone is rarely curative. Therefore, a correct preoperative diagnosis, choosing the suitable surgical technique, considering the infiltrative nature of the cyst, intraoperative prophylaxis to reduce spillage, and posterior surgical approach for treatment are of crucial importance in preventing recurrence.

## Data Availability

All data generated or analyzed during this study are included in the article.

## Abbreviations

CE: Cystic echinococcosis
CT: Computerized tomography
MRI: Magnetic resonance imaging
R: Rib
